# Extracorporeal Shockwave Treatment as Additional Therapy in Patients with Post-Stroke Spasticity of Upper Limb—A Narrative Review

**DOI:** 10.3390/jcm13072017

**Published:** 2024-03-30

**Authors:** Michał Starosta, Klaudia Marek, Justyna Redlicka, Elżbieta Miller

**Affiliations:** Department of Neurological Rehabilitation, Medical University of Lodz, Milionowa 14, 93-113 Lodz, Poland; klaudia.marek@umed.lodz.pl (K.M.); justyna.redlicka@umed.lodz.pl (J.R.); elzbieta.dorota.miller@umed.lodz (E.M.)

**Keywords:** stroke, rehabilitation, extracorporeal shockwave therapy, spasticity, upper limb, motor function

## Abstract

Stroke is a severe injury of the central nervous system (CNS) and one of the leading causes of long-term disability and mortality. One of the main symptoms of neurological diseases is spasticity. This is defined as a motor condition characterized by a velocity-dependent increase in tonic stretch reflexes with exaggerated tendon jerks and resulting in the hyperexcitability of the stretch reflex. Rehabilitation after a stroke is focused on relearning lost skills and regaining independence. Many new methods in neurorehabilitation have been introduced. This review concentrates on the current evidence for extracorporeal shockwave therapy (ESWT) as a noninvasive alternative to treat spasticity. We present the effect of EWST and radial EWST interventions to post-stroke patients with spasticity in the upper limb. Our collected data suggest that different parameters of shockwaves can be used to achieve functional improvementsin the upper limb after a stroke. Our accumulated data imply that ESWT is safe and can be used for pain relief, reduced muscle tension, and an increased range of motion. According to many studies, complications after shockwave treatment are infrequent. Transient complications after shockwave therapy (ESWT) include redness, tingling, pain, and bruising. We reviewed clinical trials that present the possible benefits in upper-limb function after shockwave therapy for post-stroke patients. In this article, we used many database search engines, including PEDro. In the stroke rehabilitation literature, a key methodological problem is the design of double-blind studies, which very often are not feasible.

## 1. Introduction

Stroke is the second most common cause of death and the leading cause of disability worldwide [1]. According to analyses, more than 50 million people worldwide havepost-stroke disabilities [2]. By 2047, it is estimated that the number of people with a stroke diagnosis in the European Union will increase by 27%. The reason for this upward trend is an aging population and improved survival rates [3]. A common complication after stroke is spasticity [4]. Spasticity was first described by Lance. It is defined as a motor condition characterized by a velocity-dependent increase in tonic stretch reflexes with exaggerated tendon jerks and resulting in the hyperexcitability of the stretch reflex [5]. Spasticity is related to upper motor neuron syndrome (UMNS) [6]. This complication often occurs in neurological diseases, including after a stroke [7]. This condition reduces patients’ quality of life through complications such as contractures, pain, reduced joint range of motion, reduced motor function, reduced mobility, and skeletal deformities [8,9]. It affects about 17–43% of chronic stroke survivors [10]. Lack of movement as a result of paralysis, along with rigidity caused by spasticity in the limb, accelerates the formation of contractures [11]. According to Wissel et al. (2010), the point at which spasticity develops is variable, but it often occurs within the first 6 weeks after a stroke in 25% of patients and affects the elbow and wrist [12]. The incidence of spasticity increases with the length of time after a stroke’s onset [10]. Post-stroke patients often show the stereotypical pattern of upper-limb impairment: adducted arm and internal rotation of the arm, bent elbow, pronation of the forearm, wrist flexion, and clenched fist. This leads to a loss of coordination between joints and joint control [13]. The item described is called the Wernicke-Mann position [14].

To better understand spasticity, the term muscle hypertonia is important. This is a general term for the increased resistance of joints to passive stretching movements. A distinction is made between neuronal and non-neuronal components, and spasticity mainly has a neuronal origin [15]. One of the main factors contributing to the symptom of muscle hypertonia in neurological disorders is the hyperactivity of spinal motor neurons [16]. There is no single definition of spasticity. The term is also commonly used as a general description of the clinical phenomenon of increased resistance to passive stretching [15]. An expanded 2005 definition proposed by Pandian et al. (2005), indicates that spasticity is a disorder of sensorimotor control. The symptoms that accompany it are temporary or permanent, such as involuntary muscle activation [17]. The new terminology refers to the increased resistance of joints and limbs as well as amovement disorder [15]. The phenomenon of spasticity has many complex and diverse aspects in its definition, which contributes to the lack of a clear consensus in the literature. Some definitions can be problematic when used insomesituations of motor disorders in post-stroke patients, due to various complications like muscle weakness and abnormal reflex reactions [18].

Despite the wide range of proposed treatments, rehabilitation programs, and therapies, spasticity remains a problem [19]. Pharmacotherapy for the treatment of spasticity usually includes drugs like baclofen, tizanidine, or diazepam. Their actions focus on the central nervous system, which leads to a reduction in the muscle tone of excessively tense muscles. Pharmacological therapy is not without its drawbacks. Medications can cause systemic side effects that affect the patient’s daily functioning, such as drowsiness or lethargy [20]. One of the most used pharmacologic therapies for treating focal spasticity is botulinum toxin type A (BoNT-A). Important factors to consider are having a well-established treatment goal, the selection of the muscle injection site, the dose and method of administration, and muscle structure [7]. The mechanism of the protein neurotoxin is to selectively inhibit the release of acetylcholine at the neuromuscular junction [21]. Side effects include hematoma at the injection site, weakness of the limb [22], pain at the injection site, dysphagia, and fatigue [23,24]. Complications after the procedure occur most often when the dose is not optimally adjusted. With multiple injections, neutralizing antibodies can form [22].

There are many options for non-pharmacological interventions to treat spasticity. These include passive movements and stretching, direct current stimulation, transcutaneous electrical nerve stimulation, vibratory stimulation, ultrasound, acupuncture, orthoses, transcranial magnetic stimulation, thermotherapy, and cryotherapy [25]. Although there are many therapeutic approaches, there is a lack of high-quality scientific evidence for most of the above-mentioned interventions [26].

**Figure 1:**
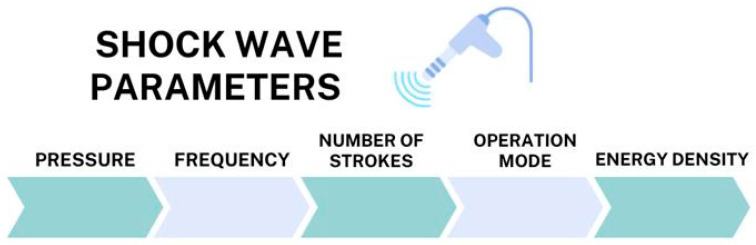
Shockwave parameters.

In the past decade, a non-pharmacological method gaining popularity in reducing muscle spasticity after a stroke is extracorporeal shockwave therapy (ESWT). This term refers to a treatment with a sequence of acoustic pulses characterized by a high peak pressure (100 MPa) and rapid pressure rise (<10 ns) and transmitted by a selected generator to a specific target area with an energy density in the range of 0.003–0.890 mJ/mm^2^ (Figure 1) [27]. Extracorporeal shockwave therapy (ESWT) is considered a noninvasive alternative for the treatment of spasticity. Publicly available data indicate that ESWT is a safe method and can be used to relieve pain, reduce muscle tension, and increase one’s range of motion.

There are two types of shockwave generators used in clinical practice: focused (FSWT) and radial (rEWST). The two types differ in their physical properties of energy and energy propagation [28]. Focused shockwavescan be produced by electrohydraulic, electromagnetic, and piezoelectric generators. It is a more invasive intervention, due to its rapidly increasing pressure and depth of action of up to 12 cm [29]. Radial shockwaves are produced by a pneumatic source placed inside a generator in the device [30]. The greatest amount of energy is concentrated at the tip of the probe, which is most often in the form of a gun, which is then transmitted deep into the tissue [31]. The depth of penetration into the tissue is less than in the focused type and is up to 3–4 cm. The smaller level of penetration is better tolerated during the procedure by patients [32]. Treatment with radial shockwave therapy (rEWST) can cause less damage to the skin and soft tissues [31], which is reflected in the low incidence ofcomplications after therapy. According to a meta-analysis by Guo et al. (2017), complications after shockwave treatment are rare [33]. Transient complications after shockwave therapy (ESWT) include redness, tingling, pain, and bruising [34]. Shockwave treatment was first performed in 1980 to treat kidney stones [35]. Shockwave therapy (ESWT) has been successfully used in the field of orthopedics for more than 20 years. The most common conditions involve tendons and musculoskeletal pathologies [30].

## 2. Mechanism of Action of Radial Shockwaves

**Figure 2:**
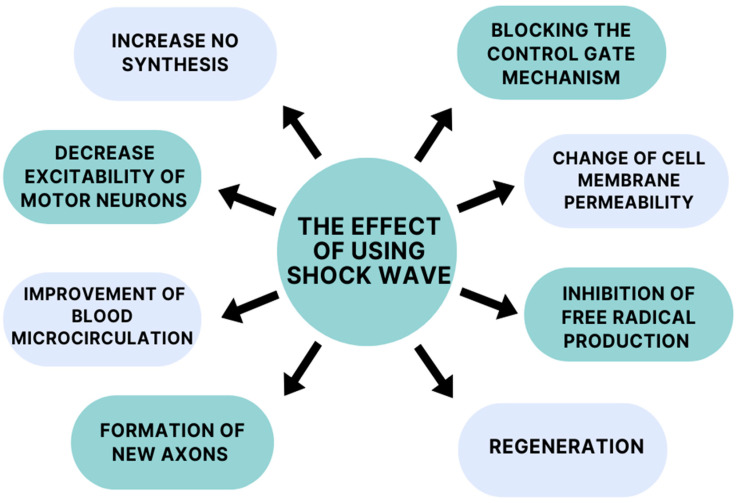
Mechanisms of action of shockwaves.

The mechanism of action of extracorporeal shockwave therapy remains incompletely understood, but it appears to demonstrate a broad spectrum of action (Figure 2). One mechanism of action of ESWT is to increase nitric oxide (NO) synthesis. This compound acts on both the peripheral and central nervous systems. In the case of the central nervous system (CNS), it affects one’s physiological functions, improving synaptic plasticity and neurotransmission. Activity in the peripheral nervous system is relatedtothe increased neovascularization of muscles and tendons [36,37]. NO is a desirable compound due to its anti-inflammatory properties. According to Mariotto (2005) et al., a molecular mechanism of anti-inflammatory action has been observed during ESWT (extracorporeal shockwave therapy), involving the tyrosine defosphorylation of endothelial nitric oxide synthase (eNOS), the successive augmentation of NO production, and the inhibition of nuclear factor kappa B (NF-κB) activation [38]. Kenmoku (2018) et al. and Jia (2020) at al. suggest in their studies that shockwave treatment can temporarily reduce acetylcholine at the neuromuscular junction, leading to a reduction in muscle spasticity [39,40].

Daliri et al. suggests in their study that shockwave therapy has a role in altering motor neuron excitability. Patients received one sham ESWT session during their study, followed by one active ESWT session 1 week later. The real session helped reduce patients’ wrist flexor spasticity and alpha motor neuron excitability. The excitability of motor neurons was measured by the neurophysiological Hmax/Mmax ratio via electromyography (EMG) [41]. According to one theory, a decrease in alpha motor neuron excitability can be caused by tendon pressure [42]. Goertz (2012) et al. used an extracorporeal shockwave treatment to accelerate the healing process in a burntmouse model. They found that the shockwaves had a positive effect on a number of burn-wound-healing parameters, in particular, thoseinregard to angiogenesis and leukocyte behavior. The number of rolling and sticking leukocytes increased, which improved the mice’s metabolism. These results imply that shockwave treatment leads to an increase in the efficiency of the tissue regeneration process by accelerating blood microcirculation and tissue rheology [43]. The basis for the initiation of the above studies was the previous confirmation by Kuo (2007) et al. of accelerated tissue repair in acute and chronic wounds with extracorporeal shockwave therapy. Furthermore, the findings presented by Link (2013) et al. also suggest the improved healing of distal wounds in horses. The reason for this is a reduction in granulation tissue production induced by the inhibition of TGF-β1 (transforming growth factor-β1) [44,45]. Kenmoku et al. (2012) conducted an extracorporeal shockwave study on rabbits. According to the researchers, the application of extracorporeal shockwaves to their muscles induced the transient dysfunction of nerve conduction at neuromuscular junctions. They found there was degeneration and a reduced number of acetylcholine receptors in the muscles, which contributed to the altered functional state. The low number of receptors is not a permanent effect; they rebuild in a short period of time. This is a possible reason for the short-term effect of extracorporeal shockwave therapy [46,47].

Neuronal effects after extracorporeal shockwave therapy have also been considered. A study by Lee et al. (2015) tells us that the early application of extracorporeal shockwaveincreases the expression of neurotrophin-3 and neurotrophin-3 mRNA. In addition, daily therapy sessions facilitated the activity of macrophages and Schwann cells, which affect neuronal survival and regeneration [48]. As we know, extracorporeal shockwave therapy reduces patients’ pain sensations. It causes the inhibition of pain fiber conduction by a altering cell membrane permeability and blocking the control gate mechanism due to the hyperstimulation of the peripheral nervous system [49]. Moreover, it shows a protective effect on neurons, due to the inhibition of the production of free radicals that lead to neuronal deformation. They positively influence the regeneration of damaged axons by stimulating one’s metabolism and the prevention of free radical damage, which is generated by axonotmesis. Extracorporeal shockwave therapy can effectively regenerate and redistribute sensory and motor fibers after a brain injury such as a stroke [50]. Many researchers have tested and applied radial shockwave treatments successfully to post-stroke patients with spasticity in the upper limb. They have reported pain relief, reduced muscle tension, and an increased range of motion [51,52,53].

### 2.1. Musculoskeletal Diseases and Spasticity

The mechanism of action of shockwaves in musculoskeletal disorders is related to multiple pathways of physiological responses, which are characterized by a final process of mechanotransduction. This is a physical-biological action during which shockwaves pass through the tissue causing high-pressure gradients [54]. Mechanotransduction affects the regulation of basic cellular functions and metabolism, such as migration, proliferation, differentiation, and apoptosis [55]. Shockwaves in musculoskeletal conditions are used to increase blood flow to the affected area by inducing healing via inflammation and secreted mediators, as well as inhibiting pain receptors and increasing angiogenesis [56]. Astur et al. (2015) performed shockwave therapy (ESWT) on patients with lower-extremity muscle injuries lasting longer than three weeks. After the treatment, they showed a reduction in pain sensations and an increase in muscle strength, allowing themtoplay sports again. To date, many biochemical studies on the effects of shockwaves on muscle tissue have been performed on mouse models in vitro and in vivo [57]. A study by Mattyasovszky et al. (2018) provides evidence of the potential of extracorporeal shockwave (rESWT) interventions to modulate the biological functions of primary human muscle cells. Shockwave treatments can affect the viability of human skeletal muscle cells and regulate the gene expression of muscle cell proteins in vitro, which can be explainedby the regenerative process [58]. Effective regeneration was observed on torn stage III muscles treated with rESWT in mice. Significant improvements in the muscle markers MyoD and myosin were observed. The presence of myosin gene expression indicated newly formed muscle fibers. This was confirmed by hematoxylin and eosin staining. Seven days after muscle injury, the number of mononuclear cells decreased, making it possible to visualize muscle fibers [59]. Speaking of muscle system regeneration, it is worth mentioning satellite cells, which are responsible for restoring function and producing a large number of new muscle fibers [60]. After anESWTintervention, the regeneration of skeletal muscle tissue is stimulated, resulting in accelerated repair processes. This is related to the increased number of satellite cells after ESWT therapy [61]. Spasticity, which is a neurological and muscular problem, is associated with the continuous stimulation of the neuromuscular plate. The effect of ESWT is similar to that of botulinum toxin A injection. In both cases, there is the destruction of endplates at neuromuscular junctions [62]. New research by Wu et al. (2019)points to the effectiveness of ESWT in treating calcified musculoskeletal structures. Functional improvements and reductionsin pain sensations in patients have been found [63]. An extensive review by Simplicio et al. (2020) demonstrates the use of shockwaves to trigger regenerative processes in the treatment of musculoskeletal disorders, including osteoarthritis [64]. In bone tissue after ESWT, the bone differentiation of stem cells and an increase in vascular endothelial growth factor (VEGF) expression are noted [65,66]. In cartilage tissue, only an increase in VEGF is noted [67]. Different tissues, whether nerve, muscle, or cartilage, respond differently to the same ESWT mechanical stimulus. Many mechanisms of ESWT remain unexplained [62].

### 2.2. Wound Healing

In aesthetic medicine, ESWT procedures are most commonly performed with the goal of treating cellulite and excess fat as well as body contouring [68]. The mechanisms used in aesthetic medicine are rooted in wound healing and regeneration. Cells respond to the indirect effects of the shockwave cavitation phenomenon, inducing changes that are beneficial in the treatment of cellulite, such as increasing local circulation and stimulating collagen production, leading to the restructuring of the dermis and epidermis, thereby restoring connective tissue elasticity and improving skin texture [68]. The phenomenon of cavitation can be explained as the formation of gas microbubbles in the fluids of biological tissues [69]. These are equally important processes in the healing of chronic wounds and burns. The first piece of supporting evidence for the healing of burn wounds and their complications usingESWTwas published in 2005 [70]. In the case of burns and hypertrophic scars following burns, the mechanotransduction process affects the transport of fibroblasts into the scar tissue, regulating the production of the molecules transforming growth factor-beta (TGF-β1), Smad, fibronectin, or collagen types I and III [54,71,72]. In addition to the process of angiogenesis, shockwavesalso affect the behavior and number of leukocytes. A study by Goertz et al. (2012) conducted on the ears of hairless mice demonstrates shockwave therapy accelerated angiogenesis, improved blood flow, and increased the leukocyte count [43]. Shockwave treatments may have a beneficial effect on the nature of hypertrophic scarring after a burn. The results of a study by Lee et al. (2021) report that ESWT interventions led to a significant reduction in scar erythema and an increase in sebum levels [73]. Changes also extend to the skin’s protective function in the stratum corneum barrier and the faster development of a uniform granulation layer [74,75]. During ESWT therapy, collagen fibers may be broken down, with consequences in terms ofscar remodeling [76]. ESWT treatments may be a promising treatment for patients with existing leg ulceration refractory to other treatments. In the results section of their study, Aschermann et al. (2017) noted morphological changes and the increased migration of keranocytes. Increased expression of the following genes responsible for cell cycle regulation was found: CCNA2, CCNB1, and CCNB2. In addition, increased secretion of pro-inflammatory cytokines responsible for accelerating wound healing and the pro-angiogenic activity of endothelial cells was noted [77].

## 3. Materials and Methods

The material collected for this review was sourced from the following databases: PubMed, PubMed Central, Cochrane Library, Medline, Embase, Web of Science, SCOPUS, and PEDro. Search topics included the following:

- Radial shockwave intervention in spasticity;
- Spasticity after stroke;
- Radial shockwave;
- Rehabilitation in spasticity;
- Stroke and shockwave.

**Figure 3:**
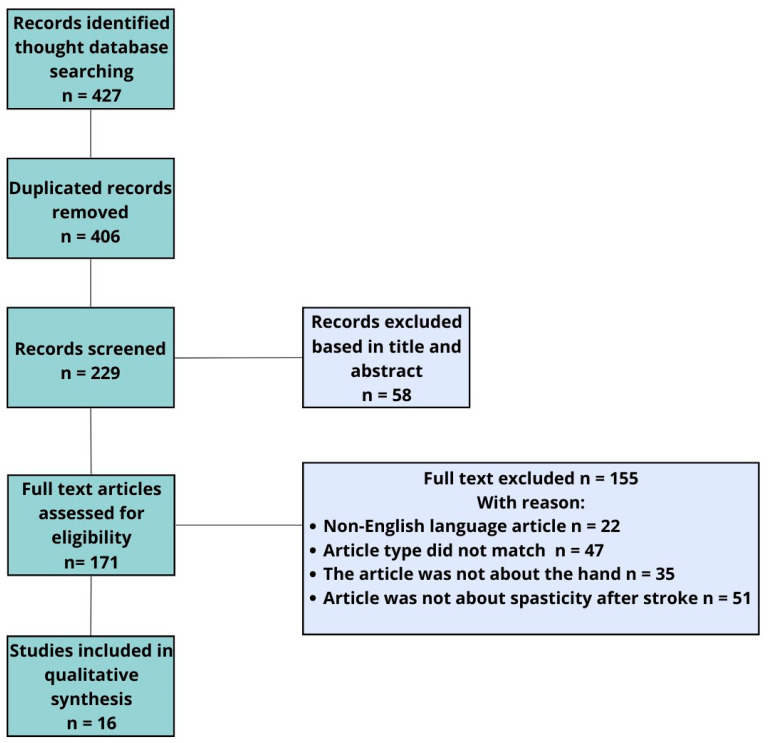
Flowchart of study selection.

In addition to the main topics, ESWT and rEWST acronyms were also searched. A total of 71 articles were analyzed. In this study, we allowed the inclusion of articles mainly from the last 18 years. After removing duplicates, we qualified 14 articles and additional 2 articles found by performing a manual search of reference lists. We eliminated articles published in a language other than English, full-access articles, articles for which only the abstract was available, studies involving the lower limb, and studies combining several investigational therapies simultaneously. For this reason, only 16 studies that met all these requirements were accepted and included in our quality synthesis (Figure 3).

## 4. Benefits of Extracorporeal Shockwave Therapy (ESWT) forStroke Patients—Results

Motor dysfunction is mostly unilateral and is related to the location and severity of a brain injury [78]. Complications of spasticity include contractures, a decreased range of motion, decreased motor function, and pain [9]. Post-stroke patients often show increased muscle tone. This symptom develops in patients after a stroke within the first 6 weeks. This abnormal pattern affects both the lower and upper limbs. In the case of the upper limb, the biggest changes involve the elbow and wrist [10]. The stereotypical pattern of upper-limb impairment is an adducted arm and internal rotation of the arm, bent elbow, pronation of the forearm, wrist flexion, and clenched fist [13].

Extracorporeal shockwave therapy (ESWT) is amethod that is gaining popularity to decrease muscle spasticity after a stroke. ESWT is used to improve the motor function of the upper limb. Treatment with ESWT involves asequence of acoustic pulses characterized by a high peak pressure (100 MPa) and rapid pressure increase(<10 ns) and transmitted by a selected generator to a specific target area with an energy density in the range of 0.003–0.890 mJ/mm^2^ [27]. In general, most studies present models of therapy with differences in terms oftreatment, treatment area, application, frequency, pressure, number of shots, and bullet size.

**Figure 4:**
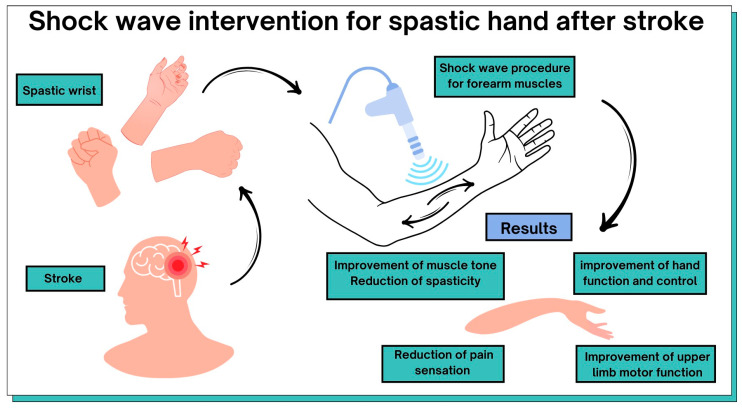
Graphical representation of the results of this review.

The most common definition of spasticity is explained as a velocity-dependent increase in muscle tension due to the presence of enhanced stretch reflexes. Spasticity significantly limits patients’ function and mobility, which can worsen long-term disabilities [79]. Extracorporeal shockwave therapy is a non-invasive tool for the treatment of post-stroke motor dysfunction in the upper limb. Table 1 and Figure 4 summarize the possible benefits of ESWT in post-stroke patients. All included original articles were evaluated on the PEDro scale, which includes 10 items corresponding to their internal validity and interpretability to assess their methodological quality. The average methodological quality score of RCT reports is 6.4 points on the 0–10 PEDro scale.

## 5. Discussion

Spasticity can reduce post-stroke patients’quality of life, interfere with their ability to be independent, and lead to the development of depressive symptoms [93]. Treatment and rehabilitation in a patient with existing spasticity can be challenging [94]. Topical invasive treatments and medications often exhibit side effects and adverse reactions, limiting their effectiveness [95]. Recent studies, as well as systematic reviews and meta-analyses, indicate the positive effects of radial shockwave intervention. This treatment is safe and itseffects are impressive. Itreducesthe degree of spasticity, decreasesthe intensity ofpain, improvesthe motor function of the affected limb, and increases patientindependence [7].

The purpose of this systematic review was to identify current scientific evidence on the use of an intervention—extracorporeal shockwave therapy (ESWT)—as a non-invasive alternative for treating spasticity in post-stroke patients. We analyzed 16 scientific studies conducted between 2005 and 2023. As many as 14 studies involved patients after an ischemic stroke. Only one study, that by Guo et al. (2019), gathered a study group of more than 100 patients, more precisely, 120 patients [53]. The large majority of studies involved a smaller number of patients; the number ranged from 20 to 60 patients [29,32,34,80,81,82,83,85,88,90,91,92]. For oursystematic review, we also qualified a case study conducted on one patient [89]. To check upper-limb recovery, the researchers mainly used the Fugl-Meyer Assessment for Upper Extremity (FMA-UE) [29,34,53,85,88,91,92] and in some individual cases, the Action Research Arm Test (ARAT) was used [88]. Muscle tension and spasticity status weretested using the Modified Ashworth Scale (MAS), Ashworth Scale (AS), Tardieu Scale, and Modified Tardieu Scale (MTS) [29,32,34,53,80,81,82,83,84,85,87,88,89,90,92]. Yuan et al. (2023) did not check their patients’ muscle tension status using the above scales [91]. Tabra et al. (2021) used an electrophysiological assessment of spasticity based on the Hmax/Mmax amplitude ratio as a reliable measure of α motor neuron excitability and spasticity [92]. The study’s authors did not use common measures of muscle tone: AS, MAS, TS, or MTS.On a case-by-case basis, the researchers used the National Institutes of Health Stroke Scale (NIHSS) to assess the severity of selected symptoms and monitor the condition of stroke patients [82], voluntary control grading (VCG) [88] to assess the voluntary control of the affected hand, the Motricity Index to assess motor impairment [92], the Modified Barthel Index (MBI) [91] and Barthel Index (BI) [32] to assess daily activities, the Disability Assessment Scale (DAS) [89] to assess the functional disability of the upper limb, and the Mini Mental State Examiation-Korea (MMSE-K) to assess cognitive function [85]. Manganotti et al.(2005) used video monitoring with a digital goniometer to assess patients’ range of motion [80].

The number of strokes applied by shockwave cartridges ranged from 1000 to 6000 strokes [34,82]. However, clinicians most often used values in the range of 1000–3000 strokes [29,32,53,83,87,90,91,92]. The shockwave pressure parameters used ranged from 1.2 to 2.5 Ba [34,84], a frequency of 4–18 Hz, and energy pulses of 0.03–0.93 mJ/mm^2^ [29,32,81,82,84]. None of the authors, except for Savevska et al. (2016), specified the parameters required for shockwave treatment. Savevska et al. (2016) were the only authorsto specify the size of the shockwave probe: 15 mm [89].

Reductions in spasticity and muscle tension were noted in every study analyzed. Three studies noted an immediate effect after oneshockwavesession [32,81,88]. There is no clear consensus on the durability of ESWT treatment. Manganotti et al. (2005) [80] points out that thiscan be up to 12 weeks after treatment, while someauthors indicate the spasticity reduction is maintained4 weeks after the intervention [82,84,88]. ESWT interventions for spastic muscles can have a positive effect as early as 1 week after starting [82]. However, Bae at al. (2010) [81] showed that this effect disappears after one week and does not persist after treatment (this is also true 4 weeks after the ESWT treatment in their study). Other cited positive effects of ESWT interventions include the following: the improvement of the functional motility of the affected limb [83], the improvement of hand function and control [34], the prevention of spasticity progression, the reduction in the use of oral antispasmodic medications [83], the improvement of active elevation of the hemiplegic upper limb [82], and pain relief [84]. The use of shockwaves in post-stroke patients may be effective in controlling the peripheral component of spasticity in terms of changes in muscle mechanical properties [29].Yoon et al. (2017) indicates that the intervention will be effective when the treatment is performed on the muscle bellies or muscle–tendon junctions of spastic muscles [87]. It should be mentioned that according to Li et al. (2020), shockwave interventions do not affect the swelling of limbs [84]. In their study, Senatarah et al. (2023) showed better results after ESTW interventions in patients after a hemorrhagic stroke compared to those of patients after a ischemic stroke after 4 weeks [88]. There are no clear recommendations on how many rESTW interventions should be performed to maintain the effect of reducing muscle tone and improving upper-limb function. The total number of rESWT interventions may affect clinical recovery during the treatment phase [90]. None of the studies reviewed found side effects after the ESTW treatments. The first research study on the use of shockwaves in the treatment of spasticity dates back to 2005 [80]. According to the National Library of Medicine’s PMC database, there are 427 articles (research articles, reviews, and meta-analyses) on the subject of the use ofshockwavesfor spasticity. As many as 305 papers deal with spasticity in post-stroke patients. In the future, we can expect great interest in shockwave therapy for spasticity.

The treatment of spasticity after a stroke is an interesting topic, but the area is dominated by relatively small studies without a control group. Studies should be conducted on a larger number of participants to estimate the treatment’s effect. Our study could be improved by listing whether the articles were systematic reviews.Additionally, we could have included a more detailed discussion of the limitations of the current study, including some suggestions for the future.

## 6. Conclusions

ESTW therapy can lead to positive effects in post-stroke patients: the improvement of muscle tone, reduction in spasticity, improvement of hand function and control, reduction in pain sensations, and improvement of upper-limb motor function. Radial shockwave interventions can complement rehabilitation in post-stroke patients with upper-limb spasticity. ESWT treatment is safe, with no long-term side effects. This review has provided a comprehensive overview of the benefits of ESWT forupper-limb rehabilitation of patients after a stroke. This may facilitate future treatment decisions for patients and initiate studies evaluating the use of ESWT at different times after a stroke. While therapy based on traditional neurophysiological methods is still useful, noninvasive ESWT is a promising additionthat can be applied to patients with spasticity. The degree of spasticity influences the final treatment result. Taking this into account, other therapeutic approaches require further clinical trials. Despite limited scientific evidence, our review shows that ESWT is mostly beneficial for patients. A protocol for the selection of treatment parameters should be standardized, and recommendations for the use of ESTW in post-stroke patients with spasticity should be made. Furthermore, patients’ level of spasticity should be taken into account in future clinical trials. In our review, we used the PEDroscale, which is a comprehensive tool to assess the methodological quality of the neuro-rehabilitation literature.

## Figures and Tables

**Table 1:** Potential benefits in upper-limb motor function after post-stroke shockwave therapy with a valid measure of methodological quality of clinical trials using the PEDro scale.

Study Author, PEDro Scale, Year	Stage of Stroke	ApplicationArea, Frequency, Pressure, Pulsecount, Bulletsize	Outcome, Measures	MainFindings
Manganotti et al. [80], 2005 PEDro: none	Clinical Trial, *n* = 20, Chronic	Forearmflexor muscles, Interosseusmuscles, 4700 shots, 0.030 mJ/mm^2^	Ashworth, NIHSS, Video monitoring with a digitalgoniometer	ESWT reduces hypertonia of the flexor wrist and finger muscles for 12 weeks after treatment.
Bae et al. [81], 2010 PEDro: none	Clinical Trial, *n* = 32, Chronic	Biceps muscle, Musculotendinousjunction of biceps, 1200 shots, 0.12 mJ/mm^2^, 4 Hz	Ashworth, Tardieu, Barthel	ESWT for chronic stroke patients’ spasticity of upper limbs has immediate effect. The treatment effect at the musculotendinous junction was greater than on the biceps.
Yoo et al. [82], 2008 PEDro: none	Clinical Trial, *n* = 21, Chronic	Biceps muscle, Forearmflexor muscles, 1000 shots, 0.069 mJ/mm^2^, 4 Hz	Ashworth, Tardieu, NIHSS	Patients treated with ESWT showed significant improvement in muscle tone of elbow flexor and wrist pronator after the 1st and 4th weeks. Active elevation of the upper limb with hemiplegia was significantly increased.
Brunelli et al. [83], 2022 PEDro: 5/10	Clinical Trial, *n* = 32, Subacute	Biceps muscle, Forearmflexor muscles, 2000 shots, 1.5 bar, 10 Hz	Ashworth	The early treatment of upper-limb muscular spasticity after stroke with ESWT seems to avoid progression to higher degrees of spasticity and reduce the use of oral antispasmodic medication.
Li et al. [34], 2016 PEDro: none	Clinical Trial, *n* = 60, Chronic	Biceps muscle, Forearmflexor muscles, 5500 shots, 3.0–3.5 bar, 5 Hz	Ashworth, FMA	ESWT may decrease flexor spasticity of the hand and wrist with enhancement of hand function and wrist control in patients with chronic stroke. Repetitive sessions of ESWT result in a longer-lasting and more noticeable effects and are necessary for improving functional motricity.
Li et al. [84], 2020 PEDro: 7/10	Clinical Trial, *n* = 82, Chronic	Biceps muscle, Brachioradialis muscle, 6000 shots, 1.2–1.4 bar, 18 Hz	Tardieu, Ashworth, VAS, FMA	ESWT is an effective therapy for spasticity after stroke, with lasting effects on both agonist and antagonist muscles after 4 weeks. ESWT relieved pain but had no effect on active function or swelling of the upper limbs.
Park et al. [85], 2018 PEDro: 8/10	Clinical Trial, *n* = 30, Chronic	Forearmflexor muscles, Interosseusmuscles, 4700 shots, 0.03 mJ/mm^2^	MAS, MMSE-K, FMA	Upper-extremity muscle tone wassignificantly higher in the ESWT group than in the sham group. ESWT is effective for mitigating thedecrease in muscle tone in chronic stroke patients.
Dymarek et al. [86], 2016 PEDro: 6/10	Clinical Trial, *n* = 20, Chronic	Forearmflexor muscles, 1500 shots 1.5 bar, 0.03 mJ/mm^2^, 4 Hz	MAS, BI, NIHSS,	A single session of ESWT could be an effective physical treatment aimed at the reduction inupper-limb spasticity and could lead to improvement of trophic conditions of the spastic muscles in post-stroke survivors.
Leng et al. [29], 2021 PEDro: none	Clinical Trial, *n* = 27, Subacute	Forearmflexor muscles, 1500 shots, 1.5 bar, 0.038 mJ/mm^2^, 4 Hz, 15 mm	MAS, FMA	ESWT may be more effective for the peripheral component of spasticity in terms of changes in muscle mechanical properties. The optimal intervention regime of post-stroke spasticity should take into consideration both neural and non-neural factors.
Guo et al. [53], 2019 PEDro: 6/10	Clinical Trial, *n* = 120, Chronic	Forearmflexor muscles, Interosseusmuscles, 2000 shots, 2.0–3.0 bar, 8 Hz	MAS, FMA,	ESWT might be beneficial in the recovery of upper-limb spasticity in post-stroke patients. To evaluate the motor recovery, one canuse the Brunnstrom stages, not justFMA and MAS.
Yoon et al. [87], 2017 PEDro: none	Clinical Trial, *n* = 80, Chronic	Biceps muscle, 1500 shots, 0.068–0.093 mJ/mm^2^, 5 Hz	MAS, MTS	ESWT could be effective for treating chronic spasticity after stroke when applied to muscle belly or myotendinous junction.
Senarath et al. [88], 2023 PEDro: none	Clinical Trial, *n* = 53, Chronic	Teres major muscle, Brachialis muscle, Forearmflexor muscles, 1500 shots, 0.03 mJ/mm^2^, 5 Hz	MAS, VCG, FMA-UL, ARAT	ESWT could be effective for treating chronic post-stroke upper limb spasticity. The patients showed improved hand functions from the first treatment.
Savevska et al. [89], 2016 PEDro: none	Case Report, *n* = 1, Chronic	Forearmflexor muscles, Interosseusmuscles, 5000 shots, 2 bar, 10 Hz, 15 mm	MAS, DAS,	ESWT reduced the spasticity of the wrist and finger flexors after stroke. ESWT is a safe, alternative, non-invasive treatment in reducing spasticity after a stroke. This therapy opens a new field of research in the non-invasive treatment of spasticity.
Fan et al. [90], 2021 PEDro: none	Clinical Trial, *n* = 50, Chronic	Forearmflexor muscles, Biceps muscle, 2000 shots, 0.03 mJ/mm^2^, 2 bar, 8 Hz	MAS	ESWT interference may affect clinical curative during the treatment phase. Long-term pateint follow-ups after the procedure are necessary to draw conclusions.
Yuan et al. [91], 2023 PEDro: none	Clinical Trial, *n* = 30, Chronic	Upper limb, 3000 shots, 1.1–1.3 bar, 8–14 Hz	MBI, FMA	ESWT can reduce the muscle tension of patients, alleviate spasticity, promote the motor function of the upper limb, and improve the working performance of patients.
Tabra et al. [92], 2021 PEDro: none	Clinical Trial, *n* = 20, Chronic	Forearmflexor muscles, Interosseusmuscles, 2000–3000 shots, 0.25–0.84 mJ/cm^2^, 2.8 bar, 15 Hz	MAS, FMA, MI Electrophysiological assessment of spasticity by Hmax/Mmax amplitude ratio	ESWT is a valuable adjuvant treatment for spasticity of the hand and wrist in stroke patients, which can be seen in terms of improvement infunctional activity. A reduction in wrist and hand spasticity was observed.

Abbreviations: FMA—Fugl–Meyer Assessment;MMSE-K—Mini Mental State Examination –Korea; MAS—Modified Ashworth Scale; BI—Barthel Index; NIHSS—National Institutes of Health Stroke Scale; MTS—Modified Tardieu Scale; VCG—voluntary control grading; UL—upper limb; ARAT—Action Research Arm Test; DAS—Disability Assessment Scale; MBI—Modified Barthel Index; MI—Motricity Index.
